# Ranking Biochar-Fluorinated Contaminant Interactions Using Dynamic Signatures

**DOI:** 10.1021/acsomega.6c02974

**Published:** 2026-08-28

**Authors:** Calogero Librici, Paola Bambina, Ettore Madonia, Roberta Bertani, Paolo Sgarbossa, Delia Francesca Chillura Martino, Paolo Lo Meo, Pellegrino Conte

**Affiliations:** † Department of Agricultural, Food and Forest Sciences, 18998 University of Palermo, V.le delle Scienze building 4, Palermo 90128, Italy; ‡ Department of Industrial Engineering, University of Padova, Via F. Marzolo 9, Padova 35122, Italy; § Department of Biological, Chemical and Pharmaceutical Sciences and Technologies, University of Palermo, V.le delle Scienze building 17, Palermo 90128, Italy

## Abstract

Understanding how fluorinated contaminants interact with porous carbonaceous materials requires to gain insights beyond macroscopic uptake metrics, particularly when heterogeneous pore networks and surface chemistries imply multiple mobility states. Here, we introduce a dynamic-signature approach to rank biochar-fluorinated liquid interactions based on molecular mobility states probed by ^19^F fast field-cycling (FFC) NMR relaxometry. Two fluorinated model systems, a perfluoropolyether mixture (Galden HT70) and the small-molecule perfluorodecalin (PFD), were investigated as neat liquids and after contact with two activated biochars (APN1 and LP39) characterized by diverse pore architectures and surface chemistries. Inverse Laplace transform (ILT) analysis of selected relaxation decays reveals the emergence of interfacial and confinement-related heterogeneity upon biochar contact, whereas quantitative analysis of ^19^F nuclear magnetic relaxation dispersion (NMRD) profiles using a two-term rotational–translational model yields effective dynamic parameters that encode these interactions. APN1, a predominantly microporous and alkaline carbon, induces moderate perturbations consistent with partial interfacial exchange and confinement, whereas LP39, a high-surface area, mesopore-richer and acidic carbon, produces strongly dispersive NMRD signatures indicative of restricted diffusion and enhanced surface-driven relaxation. These trends are consistent across both fluorinated probes, demonstrating that the observed dynamic signatures primarily reflect biochar physicochemical properties rather than probe-specific chemistry. Overall, the results show that activated biochars can be mechanistically typified by their ability to redistribute fluorinated molecules among bulk-like, interfacial, and confined dynamic states. The dynamic-signature approach presented here provides a complementary, mechanistically grounded perspective to conventional adsorption metrics and offers a pathway toward rational selection of biochars for fluorinated-contaminant retention.

## Graphical abstract

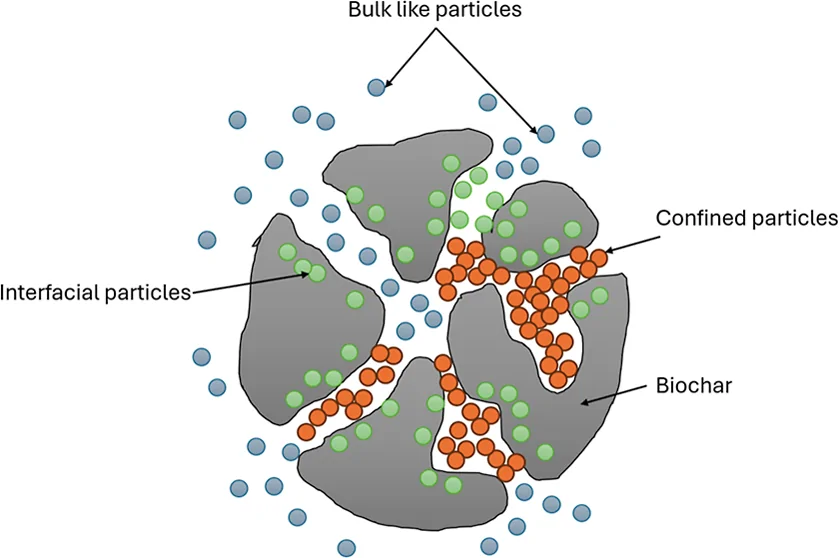

## Introduction

Per- and polyfluoroalkyl substances (PFAS) are a large class of chemically diverse anthropogenic organofluorines, characterized by exceptional chemical stability—due to extensive C–F bonding—which makes them persistent once released into the environment. A widely used structural definition (OECD)^1^ describes PFAS as fluorinated substances containing at least one fully fluorinated methyl (−CF_3_) or methylene (−CF_2_−) moiety (with a few noted exceptions). Decades of use in industrial processes and consumer products has led to their widespread occurrence in soils and water bodies, and to an evolving regulatory landscape, including stringent drinking-water standards for selected compounds worldwide.^2–4^

PFAS are most often studied through concentration-based and performance-based approaches, namely: analytical monitoring (typically LC–MS/MS for targeted PFAS), sorption experiments (batch isotherms/kinetics), and transport tests (column breakthrough) that quantify removal or retardation under specific water or soil conditions.^5–8^ In engineered treatment, adsorption-based technologies (especially activated carbon and ion-exchange resins) together with high-pressure membranes are among the most established options for PFAS-impacted waters.^5–10^ These methods, though essential, often provide limited insights into how fluorinated molecules partition between “bulk-like” and interfacial/pore domains, how heterogeneous microenvironments develop within porous sorbents, and how such heterogeneity controls apparent kinetics and reversibility.^11–15^ This mechanistic gap is particularly relevant when short-chain PFAS and complex matrices (salts, dissolved organic matter, cocontaminants) weaken or complicate adsorption behavior.^16–19^

Biochars, i.e., carbonaceous materials obtained by thermochemical conversion of biomass under oxygen-limited conditions, have emerged as a potentially scalable sorbents for contaminant management in environmental applications, including water polishing and soil remediation.^20^ Their properties can be widely tuned through feedstock selection and processing conditions, which jointly determine their aromatic character, surface functionality, mineral/ash content, and pore architecture.^20^ A growing body of work reports on PFAS uptake by biochar and biochar-derived composites, with adsorption trends depending on the PFAS structure, and on biochar surface chemistry and texture as well. Notably, performances improvements can be often observed upon applying activation/functionalization strategies on the carbonaceous pristine material.^21–23^ However, because of biochar intrinsic heterogeneous microscopic architecture, macroscopic uptake alone cannot reveal whether fluorinated species remain largely “bulk-like” dynamically slowed at external surfaces or otherwise undergo partition into confined pore domains. These distinctions indeed matter for mechanistic interpretation and for designing sorbents that truly retain fluorinated contaminants.^24^

Here, we address these problems by introducing a dynamic-signature approach to rank biochar-fluorinated contaminant interactions based on the *mobility states* experienced by fluorinated molecules in contact with carbon materials. “Dynamic signatures” are extracted from ^19^F fast field-cycling (FFC) NMR relaxometry, which combines the intrinsic selectivity of ^19^F NMR for fluorinated species with relaxation-dispersion (NMRD) sensitivity to molecular motion over multiple time scales.^25^ Fluorinated liquids have, in fact, been employed as ^19^F-selective dynamic tracers in NMR relaxometry studies of geological and other porous media since the early 2000 s, owing to their favorable relaxation sensitivity and their general absence from natural matrices; however, such studies have remained comparatively scarce relative to the extensive water- and hydrocarbon-focused surface-relaxation literature.^26^

Inverse Laplace transform (ILT) analysis further enables identification of heterogeneous relaxation populations as distinct components.^25^ Using two complementary fluorinated model systems—a perfluoropolyether mixture (Galden HT70) representing polymeric, ether-containing fluorinated chemistry, and perfluorodecalin representing a compact perfluorinated small molecule—we compare ^19^F NMRD profiles and ILT distributions before and after contact with selected biochars that differ in texture and pore characteristics. Here, Galden HT70 and perfluorodecalin are used as neutral fluorinated probe liquids (operational proxies) to interrogate confinement/interfacial dynamics in porous carbons for mechanistic ranking. They are not intended to represent the structural diversity or regulatory target list of environmentally monitored PFAS. In particular, both probes are electrically neutral and lack the ionic, amphiphilic headgroups (e.g., carboxylate, sulfonate) characteristic of regulated PFAS such as perfluorooctanoic acid (PFOA) and perfluorooctanesulfonate (PFOS), which may introduce additional, charge-driven interaction mechanisms with biochar surfaces not captured by the present neutral-probe approach.

The specific objectives of this study are (i) to establish reference dynamic signatures for the neat, fluorinated probes; (ii) to quantify biochar-induced changes in relaxation dispersion and ILT-derived population heterogeneity consistent with interfacial/pore partitioning and dynamic immobilization; and (iii) to translate these changes into comparative metrics that enable mechanistic ranking of biochar-fluorinated contaminant interactions. In doing so, the study provides a mechanistic basis for evaluating biochar-derived carbons as candidate retention media for fluorinated contaminants, complementing conventional concentration-based adsorption assessments.

## Materials and Methods

### Fluorinated Compounds

Galden HT70 and perfluorodecalin were used as fluorinated reference liquids for ^19^F FFC NMR relaxometry measurements. Galden HT70 is a perfluoropolyether (PFPE) commercial fluid supplied as a mixture of oligomers, commonly represented by a polymeric composition of the type ${\left[CF\left(CF_{3}\right)CF_{2}O\right]}_{x}{\left(CF_{2}O\right)}_{y}$ with a distribution of chain lengths rather than a single, discrete molecular species. The material was purchased from Stelar S.r.l. (Mede, Pavia, Italy). Perfluorodecalin (C_10_F_18_, PFD) is a chemically inert, nonpolar perfluorinated liquid and was used here as a low-molecular-weight fluorinated reference compound (a small-molecule counterpart to the PFPE fluid). The material was purchased from Sigma-Aldrich and Merck. Both liquids were used as received, without further purification.

### Activated Biochars

Two carbonaceous sorbents were investigated as follows: (i) LP39, a commercial activated carbon purchased from Jacobi, and (ii) APN1, a KOH-activated carbon prepared from spent coffee grounds.^27^ LP39 was used as received.

APN1 was produced by pyrolysis/activation of washed spent coffee grounds under nitrogen in the presence of KOH (activator/substrate ratio 1:1, w·w^–1^). In the preparation protocol, the material was heated from room temperature to the final temperature at 20 °C min^–1^ and held for 2 h under N_2_ flow (100 mL min^–1^) in a horizontal stainless-steel reactor; in the reference preparation series, the final pyrolysis temperature for APN1 was 800 °C.^27^

### Activated Biochar Chemical Characterization

The two activated biochars (LP39 and APN1) were previously characterized in detail by a multitechnique approach, including elemental analysis (CHNS) and FTIR spectroscopy, Raman spectroscopy (including band deconvolution and ID/IG metrics), X-ray diffraction (XRD; d002 spacing and crystallite stacking parameters), and X-ray photoelectron spectroscopy (XPS; surface elemental composition and chemical-state analysis). In addition, solid-state ^13^C CPMAS NMR was used to probe the carbon structural domains, while surface chemistry was quantified by Boehm titration, and the point of zero charge (PZC) was determined. Textural properties were assessed by N_2_ adsorption at 77 K (BET surface area, pore volumes, and pore-size distribution), and morphological features were investigated by environmental SEM (with embedded semiquantitative microanalysis) and, for selected samples, TEM. Numerical characterization results for LP39 and APN1 are reported in Rosson et al.^27^ and summarized here in Table 1. Throughout the discussion, we refer to those independent characterization data sets to rationalize the ^19^F FFC-NMR dynamic signatures and the resulting mechanistic interpretation of fluorinated liquid trapping.

**Table 1:** Textural and Acid–Base Properties of the Activated Biochars LP39 and APN1^c^

property	LP39	APN1
SBET (m^2^ g^–1^)^a^	1782	1200
total pore volume, *V*_t_ (cm^3^ g^–1^)	1.62	0.50
micropore volume, *V*_μ_ (cm^3^ g^–1^)	0.51	0.43
micropore fraction, *V*_μ_/*V*_t_ (%)	31.5	86.0
average pore diameter (nm)	3.64	1.67
point of zero charge (PZC)	6.7	9.5
basic sites (mmol g^–1^)	n.d^b^	1.50
carboxyl groups (mmol g^–1^)	0.49	0.05
phenolic groups (mmol g^–1^)	0.51	0.45

a SBET = specific surface area determined by the Brunauer–Emmett–Teller (BET) method.

b Not determinable.

c Note: The values reported in this Table are taken from Rosson et al.^27^

In the present study, additional solid-state direct-excitation ^13^C MAS NMR spectra were acquired (^13^C DPMAS NMR). Spectra were acquired on a Bruker spectrometer operating at 400 MHz for ^1^H (100 MHz for ^13^C) using a 4 mm wide-bore MAS probe and 4 mm zirconia rotors with Kel-F caps. Samples were spun at 8 kHz and maintained at 298 K. Spectra were recorded using the Bruker hpdec.av pulse program (single-pulse direct excitation with high-power ^1^H decoupling). The ^13^C 90° pulse length was 5 μs; spectral width was 25,252.5 Hz; acquisition time was 0.032 s (DW = 19.8 μs, TD = 16,384). ^1^H decoupling during acquisition was applied using a TPPM15 scheme. Recycle delays of 60 s and 128 scans were used. An exponential line broadening of 100 Hz was applied prior to Fourier transform, followed by automatic baseline correction. Chemical shifts were referenced to TMS using adamantane as an external standard.

### ^19^F Fast Field Cycling (FFC) NMR Relaxometry

FFC-NMR measurements were performed on a Stelar SmarTracer benchtop relaxometer (Stelar, Mede, PV, Italy). Neat-liquid measurements were carried out by loading 1.0 mL of Galden HT70 or perfluorodecalin (PFD) into 10 mm NMR tubes (NMR grade). For biochar-contact samples, 1.00 g of activated biochar (APN1 or LP39) was combined with 2.50 g of the fluorinated liquid (biochar/liquid mass ratio = 1:2.5, w/w), transferred into 10 mm NMR tubes, and sealed prior to measurements. Liquid densities were 1.68 g mL^–1^ for Galden HT70 and 1.91 g mL^–1^ for PFD, corresponding to liquid volumes of 1.49 mL (Galden) and 1.31 mL (PFD) in the 2.50 g mixtures. The solid–liquid mixtures were left in contact overnight (∼12–16 h) at room temperature before each analysis. No excess liquid was removed prior to measurements. For each sample, ^19^F longitudinal relaxation times T_1_ were acquired over a Larmor-frequency (ν_L_) range of 0.01–10 MHz using prepolarized (PP) and nonpolarized (NP) sequences at a fixed acquisition field of 7.65 MHz, with a PP/NP crossover set at 3 MHz. Magnetization decays (PP) and recoveries (NP) were fitted by nonlinear least-squares to a single stretched-exponential function, yielding one effective *T*_1_ value per sample and frequency. Relaxation rates *R*_1_(ν_L_) = 1/*T*_1_ were then calculated to obtain nuclear magnetic relaxation dispersion (NMRD) profiles.^25^ Additional details on the two pulse sequences can be found elsewhere.^25^

### Inverse Laplace Transform (ILT) of Three Selected Decay Curves

Relaxation decays were analyzed by inverse Laplace transform (ILT) at three representative magnetic fields (^19^F Larmor frequencies: 10, 1, and 0.02 MHz), chosen to sample the high-, intermediate-, and low-field regimes within the explored range. ILT calculations were performed using the UPEN (Uniform PENalty) regularization algorithm,^28–31^ implemented in the UPEN software package (University of Bologna). Because ILT is a regularized inversion of an ill-posed problem, *T*_1_ distributions are used here as a qualitative, model-independent diagnostic of dynamic heterogeneity. In the present context, the distributions serve to assess the emergence of interfacial- and confinement-related relaxation contributions upon contact between fluorinated liquids and activated biochars. All analyses were run using the software default settings (including the default regularization parameter, β = 1).

### Two-Terms Analysis of ^19^F NMRD Profiles

NMRD profiles (*R*_1_ vs ^19^F Larmor frequency) were quantitatively analyzed using WinREX (Extra Byte srl, Castano Primo, Milan, Italy) by adopting a two-term model in which the total relaxation rate is expressed as the sum of a rotational BPP contribution and a translational BPP contribution^32^

1 $$R_{1}\left(\omega \right)=R_{1,0}+R_{1,rot}\left(\omega \right)+R_{1,trans}\left(\omega \right)$$

where *R*_1,0_ is a frequency-independent baseline term and ω = 2πν_L_ is the angular Larmor frequency. The rotational BPP term (isotropic reorientational modulation of dipolar interactions) was modeled as^32^

2 $$R_{1,rot}\left(\omega \right)=c_{rot}\left[\frac{\tau_{rot}}{1+{\left(\omega \tau_{rot}\right)}^{2}}+\frac{4\tau_{rot}}{1+4{\left(\omega \tau_{rot}\right)}^{2}}\right]$$

where τ_rot_ is an effective rotational correlation time and *c*_rot_ is a prefactor that groups the strength of the relaxation interaction (effective dipolar-coupling modulation) and geometric factors. The translational BPP term (diffusion-driven modulation of intermolecular distances and dipolar couplings) was modeled as^33^

3 $$R_{1,trans}\left(\omega \right)=c_{trans}\int_{0}^{\infty }\frac{u}{u^{4}+{\left(\omega \tau_{trans}\right)}^{2}}J_{-3/2}{\left(u\right)}^{2}du$$

where τ_trans_ is an effective translational correlation time, *c*_trans_ is the corresponding prefactor, *u* is a dimensionless integration variable, and *J*_–3/2_(*u*) is a Bessel function. In this framework, τ_rot_ and τ_trans_ should be interpreted as effective time scales capturing the dominant reorientational and translational channels over the measured frequency window, particularly in heterogeneous systems where exchange between more bulk-like and interfacial/confined environments may occur. Best-fit parameters for all systems are reported in Table 2. Goodness-of-fit was assessed using the root-mean-square (RMS) of residuals and the model/data match returned by WinREX. Given the phenomenological nature of the model and parameter interdependence, no formal statistical uncertainties are reported; the fitted parameters are therefore treated as effective descriptors whose main value lies in the consistent relative trends across systems.

**Table 2:** Best-Fit Parameters Obtained from Two-Term Rotational–Translational BPP Modeling of ^19^F NMRD Profiles for Neat-Fluorinated Liquids (Galden HT70 and Perfluorodecalin, PFD) and for Their Mixtures with Activated Biochars (APN1 and LP39), as Derived Using WinREX^a^ ^c^

system	baseline (*R*_1,0_, s^–1^)	τ_rot_ (ns)	*c*_rot_ (s^–1^)	τ_trans_ (μs)	*c*_trans_ (s^–1^)	RMS^b^	model/data match (%)^b^
bulk Galden	1.25	157	2.38 × 10^5^	23.3	0.50	0.01958	98.7
APN1 + Galden	1.16	142	7.00 × 10^5^	28.2	4.19	0.04032	98.9
LP39 + Galden	3.45	149	5.15 × 10^6^	22.3	0.61	0.17240	97.1
bulk PFD	1.10	38.9	4.92 × 10^6^	16.1	0.95	0.02436	99.3
APN1 + PFD	7.29 × 10^–12^	14.3	2.53 × 10^7^	1.23	5.45	0.03617	96.5
LP39 + PFD	3.43	255	2.23 × 10^6^	37.6	50.8	0.14180	98.1

a The model includes a field-independent baseline contribution (*R*_1,0_) and two effective dynamic terms accounting for rotational and translational relaxation contributions. Parameters are reported for comparative purposes only and are used to support a qualitative interpretation of bulk-like versus interfacial/confinement-dominated dynamics upon biochar contact.

b RMS denotes the root-mean-square of the fit residuals (dimensionless), used here as a compact indicator of goodness-of-fit; model/data match reports the percent agreement returned by WinREX.

c Note. For APN1 + PFD, the fitted baseline term (R_1,0_) converged to a negligible value (≈0) within numerical tolerance and should be interpreted as effectively zero within the uncertainty of the fit.

Physically, *R*_1,rot_(ω) is expected to arise predominantly from intramolecular ^19^F–^19^F dipolar interactions between fluorine pairs on the same molecule, modulated by overall molecular tumbling. Intermolecular ^19^F–^19^F contributions arising from the rotational reorientation of spin pairs located on different molecules are expected to be comparatively minor, owing to the longer average intermolecular F···F distances, and are therefore not explicitly resolved by the rotational term. Conversely, *R*_1,trans_(ω) accounts for intermolecular ^19^F–^19^F dipolar interactions between spin pairs on different molecules, modulated by their relative translational diffusion,^33,34^ that is, by the time-dependent change in internuclear distance as molecules diffuse past one another. The frequency-independent offset *R*_1,0_ was retained in the fitting model to account for relaxation contributions from motions too fast to be resolved within the explored Larmor-frequency window (0.01–10 MHz), such as small-amplitude librations and bond vibrations, whose associated correlation times (subpicosecond to low-picosecond range) place their dispersion well above the accessible frequency range. Within the present two-term framework, *R*_1,0_ therefore implicitly absorbs these fast, unresolved contributions rather than requiring a dedicated third dispersive term. Inclusion of *R*_1,0_ as a free parameter improved the fit quality (lower RMS, higher model/data match) relative to a baseline-free two-term model for all systems except APN1 + PFD, for which *R*_1,0_ converged to a value statistically indistinguishable from zero (Table 2, Note), indicating that fast unresolved motions contribute negligibly to relaxation in that particular system. It should be noted that more elaborate multicorrelation-time frameworks (e.g., three-τ models that explicitly separate fast intramolecular, slow interfacial, and exchange-mediated contributions) have been developed for systems dominated by strong surface interactions.^26,35^ The present two-term model was adopted as a deliberately parsimonious, comparative framework suited to the moderate perturbations observed here, rather than as a claim of full mechanistic completeness.

## Results and Discussion

### Sample Characteristics

Both LP39 and APN1 (denoted AAC-9 in Rosson et al.^27^) are highly porous activated biochars, yet they differ markedly in pore architecture and surface acid–base character, two dimensions that will be used later to rationalize the distinct ^19^F FFC-NMR dynamic signatures. As summarized in Table 1, LP39 combines very high SBET and Vt with a mesopore-richer texture, whereas APN1 is predominantly microporous (*V*_μ_/*V*_t_ ≈ 86%; micropores defined as pores with width <2 nm, mesopores 2–50 nm, and macropores >50 nm, following IUPAC recommendations^36^). Consistently, these metrics point to APN1 as a “micropore-biased” carbon where confinement effects are expected to be prominent, whereas LP39 combines a very high surface area with a more extensive mesopore network that can host a larger bulk-like fraction of the fluorinated liquid. Surface chemistry further differentiates the two materials: LP39 shows an acidic/near-neutral surface character (point of zero charge, PZC = 6.7, Table 1) and Boehm titration indicates ∼1.00 mmol g^–1^ total acidic sites (carboxylic 0.49; phenolic 0.51 mmol g^–1^, Table 1) with essentially no basic sites, while APN1 is more basic (PZC = 9.5, Table 1) with 1.50 mmol g^–1^ basic sites and 0.50 mmol g^–1^ total acidic sites (carboxylic 0.05; phenolic 0.45 mmol g^–1^, Table 1).

To complement the CPMAS ^13^C NMR data reported by Rosson et al.,^27^ we acquired ^13^C DPMAS (direct-excitation) spectra (Figure 1), in such a way to emphasize the nonprotonated, highly condensed carbon pool and help discriminate between aromatic condensation and residual aliphatic/oxygenated fragments. It can be observed that LP39 is dominated by condensed aromatic domains with measurable oxygenated acidic groups, whereas APN1 displays a more heterogeneous carbon environment (condensed aromatic plus oxygenated/aromatic and residual aliphatic fragments), consistent with its distinct surface polarity and pore architecture. This picture is consistent with independent evidence reported by Rosson et al.:^27^ the higher Raman ID/IG ratio and more graphitized character documented for LP39 support its dominance of condensed aromatic domains, whereas the lower crystallite stacking order and higher XPS-derived surface oxygen content reported for APN1 are consistent with its more heterogeneous, oxygenated carbon environment. Together, the harmonized textural and acid–base metrics outline two different “interaction landscapes” (LP39: mesopore-richer, acidic; APN1: micropore-biased, basic) that will be explicitly linked to the ^19^F FFC-NMR relaxation-dispersion signatures in the subsequent discussion.

**Figure 1:**
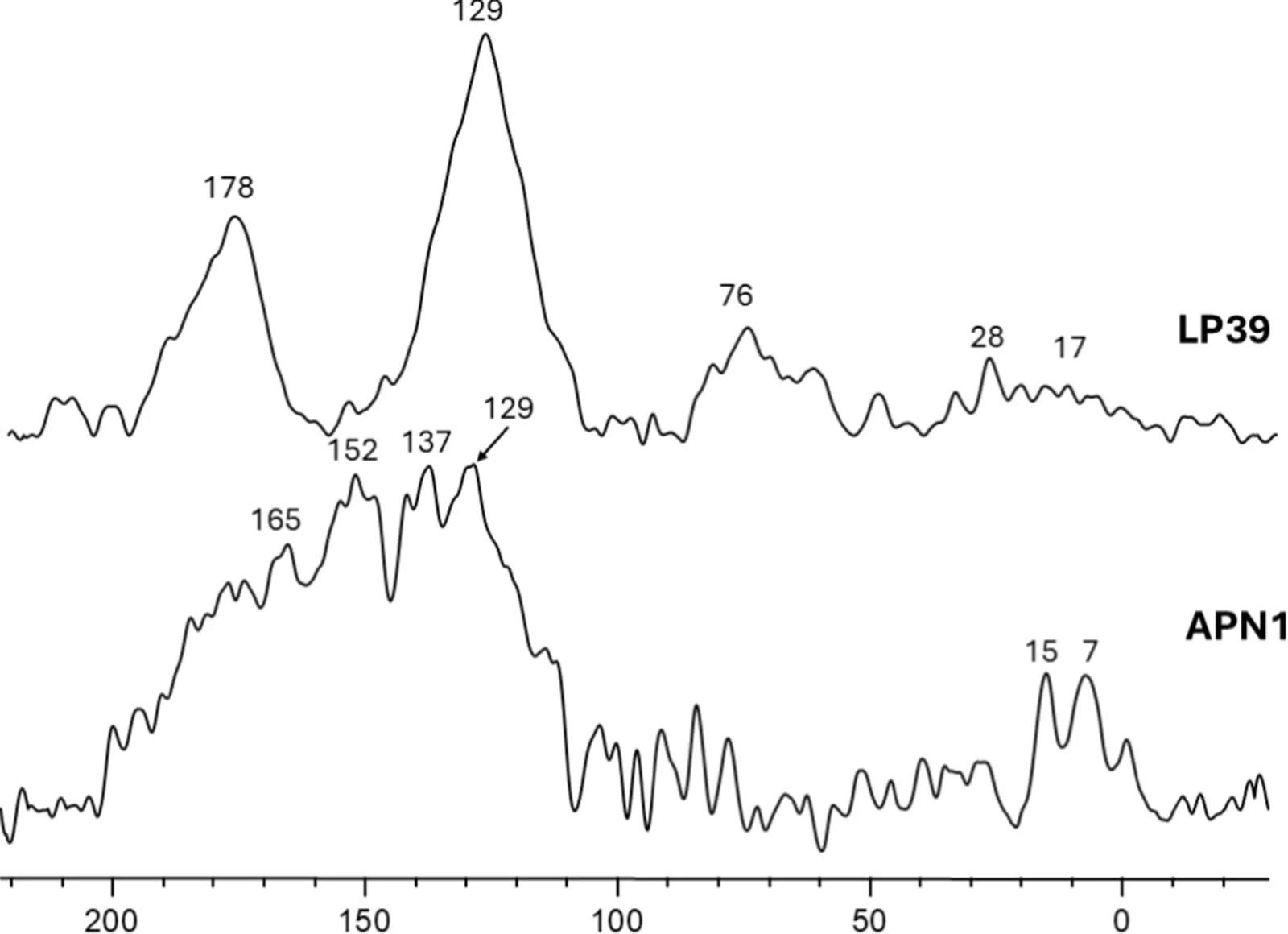
Solid-state ^13^C DPMAS (direct-excitation) NMR spectra of the activated biochars LP39 (top) and APN1 (bottom), recorded at 298 K under MAS (8 kHz) with high-power ^1^H decoupling (TPPM15). Chemical shifts are referenced to TMS via adamantane. The main resonances are assigned to carbonyl/carboxyl C (∼165–178 ppm), O-substituted aromatic/phenolic C (∼150–160 ppm), aromatic C (∼120–140 ppm), and alkyl C (∼0–40 ppm); peak positions (ppm) are indicated above the corresponding features.

### Inverse Laplace Transform of the Perfluorinated Liquids and Their Mixtures with the Activated Biochars

Inverse Laplace transform (ILT) analysis was used to qualitatively inspect the distribution of longitudinal relaxation times (*T*_1_) for Galden (Figure 2A–C) and perfluorodecalin (Figure 2D–F), both as neat liquids and after contact with the activated biochars APN1 and LP39, at three representative fields, namely, 10.0 MHz (Figure 2A,D), 1.0 MHz (Figure 2B,E), and 0.02 MHz (Figure 2C,F).

**Figure 2:**
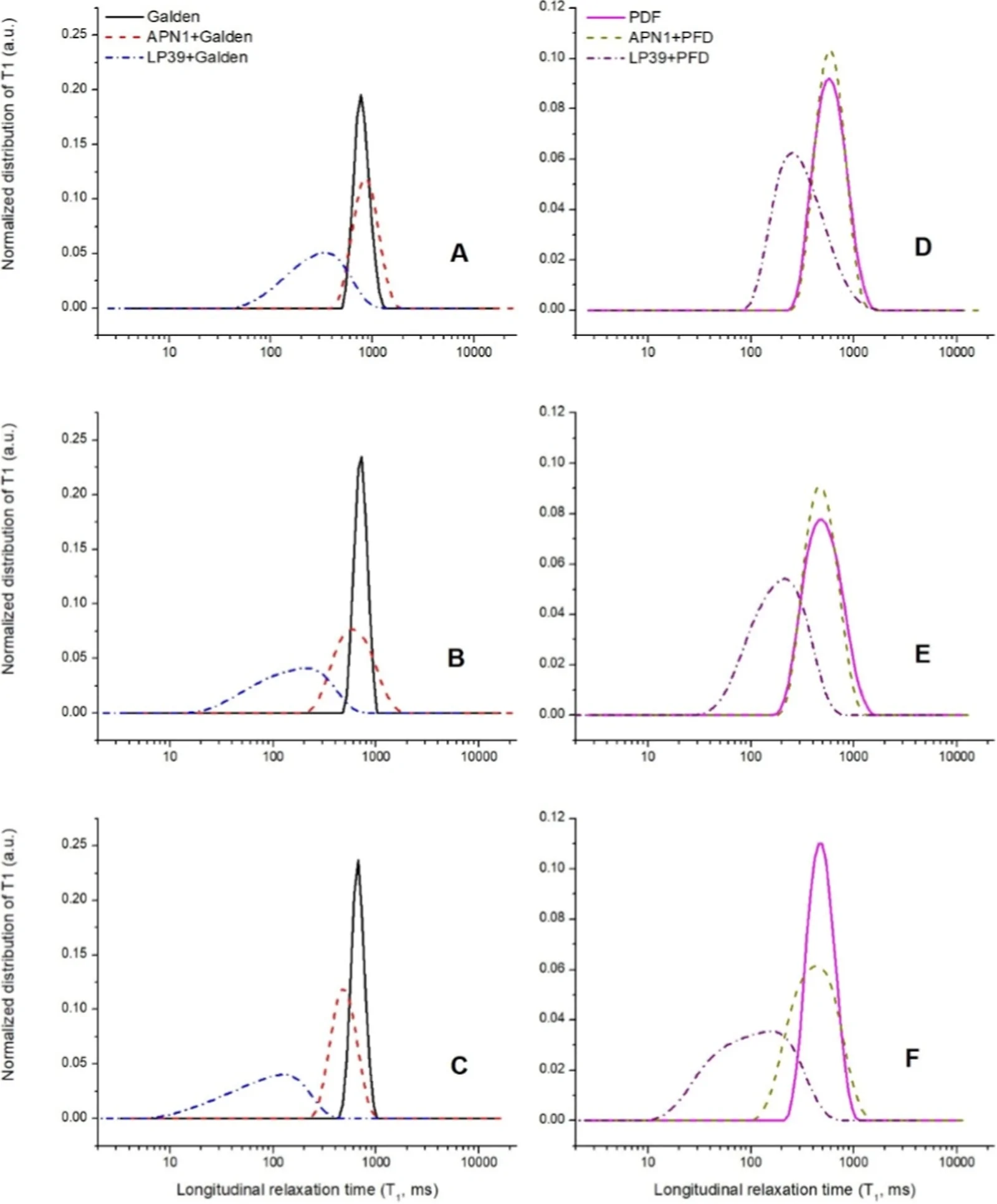
Inverse Laplace transform (ILT) distributions of longitudinal relaxation times (*T*_1_) for Galden (A–C) and perfluorodecalin (PFD; D–F), measured as neat liquids and in contact with the activated biochars APN1 and LP39 at three magnetic fields: 10 MHz (A,D), 1 MHz (B,E), and 0.02 MHz (C,F). The ILT results are shown for qualitative comparison of *T*_1_ distribution shapes and field-dependent trends. A detailed discussion of the observed behavior and its implications is provided in the main text.

Synoptical inspection of Figure 2A,D reveals some interesting trends. Both neat Galden and PFD show narrow *T*_1_ distribution at the three representative magnetic fields, consistent with what expected for free liquids. Interaction with the two different carbonaceous materials gives rise to distinct patterns. In the presence of APN1, *T*_1_ distributions for PFD are barely distinguishable from the neat liquid at 10 and 1 MHz, whereas modest changes can be observed at 0.02 MHz. Conversely, more apparent field-dependent modifications of the *T*_1_ distribution curves are found for Galden. By contrast, significant modifications occur in the *T*_1_ distributions for both organofluorines when interacting with LP39. Once again, larger effects can be observed for Galden than PFD. These findings consistently suggest that Galden develops a more significant field-dependent interfacial heterogeneity in its behavior upon contact with the carbonaceous material. This can be clearly attributed to the presence of the hydrogen bond acceptor oxygen atoms present in the structure of Galden (lacking in PFD). Notably, both liquids show marked deviations from the neat-liquid behavior upon contact with both APN1 and LP39, indicating that surface-driven relaxation becomes increasingly dominant at low field. A qualitatively similar low-field dispersion pattern, attributed to liquid molecules transiently bound at a solid surface, has been extensively documented for water and other liquids confined in porous media, where the field-dependent relaxation dispersion is interpreted in terms of a surface dynamical correlation time analogous to the τ_trans_ parameter used here.^26^ At the same time, LP39 seems able to induce larger interfacial/confinement-dominated contributions in the biochar-contact samples than APN1. The latter observation qualitatively justifies the use of a multicomponent description for interpreting the corresponding NMRD profiles (see below).

### Nuclear Magnetic Relaxation Dispersion Profiles

^19^F NMRD profiles provide a compact “dynamic signature” of fluorinated liquids by sampling molecular motion over multiple time scales (approximately ns−μs) through the frequency dependence of the longitudinal relaxation rate *R*_1_. In practical terms, an upward shift of an entire NMRD curve relative to a reference (e.g., neat liquid) indicates enhanced overall relaxation efficiency, i.e., more restricted or slower molecular motion on average. An increase in the curve’s field dependence (stronger dispersion) indicates that the probed motions span a broader range of correlation times, typically reflecting exchange between distinct dynamic environments such as bulk-like and surface-bound states.^26^ In this work, dynamic signatures are extracted by comparing each neat-fluorinated probe (bulk liquid) with its corresponding “on-biochar” systems, where the interaction with the porous carbon matrix can induce confinement, interfacial exchange, and heterogeneous mobility states. Quantitative analysis of all NMRD curves was performed using a two-term model (rotational BPP + translational BPP), yielding a baseline plus effective rotational and translational correlation times and prefactors (Table 2). Besides, an *R*_1,0_ baseline (also referred to as offset) term accounts for possible motions too fast to be sampled by the FFC method, within the Larmor frequency range explored (see the Materials and Methods section for a discussion of its physical origin).

Bulk perfluorodecalin (PFD) exhibits a relatively weak dispersion over 0.01–10 MHz, consistent with fast, liquid-like dynamics (Figure 3A). The profile is well reproduced by the two-term BPP framework, indicating that both reorientational (rotational) and diffusion-mediated (translational) channels contribute measurably across the explored window (Table 2). In physical terms, the rotational term captures time-dependent fluctuations of local dipolar interactions driven by overall molecular tumbling/rotational reorientation (including small-amplitude librations), while the translational term accounts for modulation of intermolecular couplings through relative 3D diffusion and diffusive encounters. Upon contact with APN1, the NMRD curve shifts to higher *R*_1_ values and shows a clearer field dependence (Figure 3B), evidencing an altered dynamic signature relative to the neat liquid. Within the two-term analysis, the APN1-loaded system is characterized by markedly shorter effective correlation times for both channels together with enhanced prefactors, consistent with a heterogeneous environment in which surface/porosity contact increases the efficiency of dipolar-coupling modulation (Table 2). It is noteworthy that these fitted times should be read as effective parameters under exchange between more bulk-like and surface-influenced domains rather than as literal “accelerated bulk” motion. Overall, APN1 induces a fair but appreciable perturbation of PFD mobility, consistent with interfacial interaction and partial partitioning into accessible pore domains. By contrast, LP39 produces a dramatic amplification of *R*_1_ and a strongly dispersive profile (Figure 3C), indicating a much more pronounced perturbation of the fluorinated liquid dynamics. The two-term model captures this behavior with longer effective correlation times (hindered orientational and translational motion) and a strongly enhanced translational prefactor, consistent with restricted diffusion and frequent close-contact configurations in a confined/heterogeneous pore network (Table 2). Taken together, the PFD dynamic signatures rank the interaction strength/confinement effect as LP39 ≫ APN1 > bulk, supporting stronger trapping and/or deeper pore partitioning in LP39.

**Figure 3:**
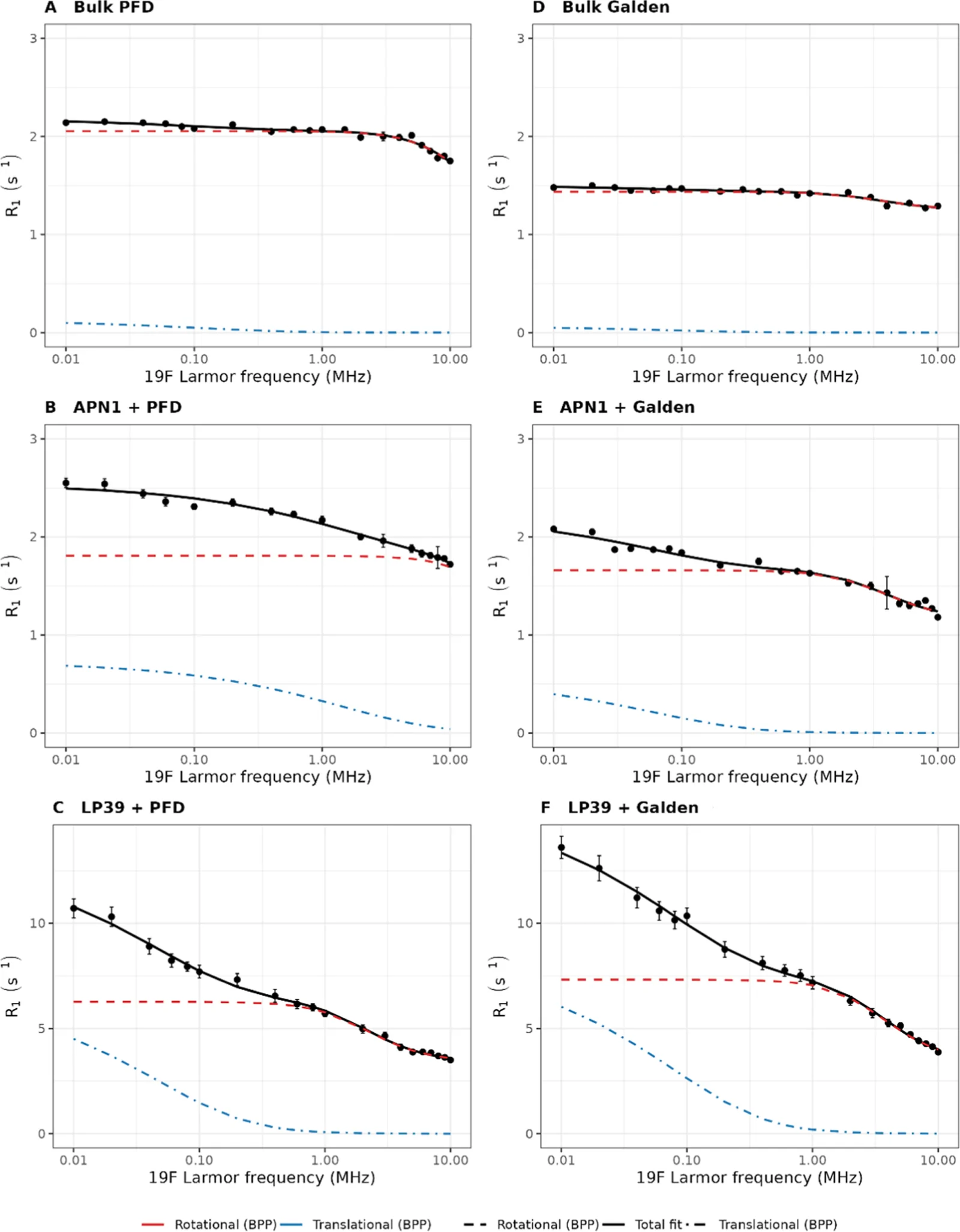
^19^F NMRD profiles (longitudinal relaxation rate *R*_1_) of perfluorodecalin (left column) and Galden HT70 (right column) as neat liquids (A,D) and after contact with activated biochars APN1 (B,E) and LP39 (C,F). Solid black lines represent the total two-term fit (rotational BPP + translational BPP); dashed red and dash–dot–dot blue lines indicate the rotational and translational BPP contributions, respectively. The *x*-axis is the ^19^F Larmor frequency (MHz, logarithmic scale). The rotational contribution includes the frequency-independent baseline term *R*_1,0_; the translational contribution is shown net of *R*_1,0_, so that the two dashed/dash–dot curves sum exactly to the total fit at every frequency.

Neat Galden shows a weak-to-moderate dispersion with *R*_1_ values comparable to (or slightly higher than) bulk perfluorodecalin in the same frequency window (Figure 3D). The two-term BPP framework again indicates combined contributions from rotational reorientations and diffusion-mediated translational encounters, yielding effective ns−μs time scales and a good global description of the profile (Table 2). Contact with APN1 produces an upward shift and modest reshaping of the NMRD curve (Figure 3E). Compared with bulk Galden, effective correlation times remain in a similar ns−μs range, whereas prefactors, especially the translational prefactor, are enhanced (Table 2). This pattern is consistent with a heterogeneous interfacial regime where diffusion-mediated encounters become more efficient contributors to relaxation, while the overall mobility remains partly bulk-like due to the fluid’s oligomeric mixture and possible dynamic exchange between environments. In turn, LP39 induces the strongest Galden dynamic-signature change, with a marked increase of *R*_1_ and a pronounced dispersion (Figure 3F). The fitted times remain broadly comparable to bulk, but the prefactors increase significantly, again with a prominent translational contribution, indicating a much larger efficiency of dipolar-coupling modulation in the porous carbon environment (Table 2). This behavior supports stronger confinement and more persistent close-contact configurations within LP39 pores. Overall, as for PFD, Galden signatures rank LP39 > APN1 > bulk in terms of confinement/interaction strength inferred from dynamics.

The effective rotational correlation times obtained here (τ_rot_ ≈ 39 ns for bulk PFD and ≈157 ns for bulk Galden) fall in a substantially longer range than those reported for small ionic-liquid ions probed by the same FFC ^1^H/^19^F relaxometry approach. Kruse et al.^37^ recently obtained internal rotational correlation times of ≈0.1 ns for a 23-atom cation and ≈0.03–0.04 ns for a 14-atom anion in an ionic liquid, with a 50% increase in the atom count corresponding to an approximately 3-fold increase in τ_rot_.^37^ Extrapolating this size-dependent trend to the considerably larger and more flexible PFD and Galden molecules studied here readily accounts for the longer τ_rot_ values obtained in the present study, thereby supporting the physical plausibility of the extracted rotational time scales.

The combined ILT and ^19^F NMRD results provide a coherent framework to relate the physicochemical properties of the two activated biochars to the dynamic states experienced by the fluorinated liquids. APN1 and LP39 define two distinct interaction landscapes: APN1 is characterized by a predominantly microporous texture (*V*_μ_/*V*_t_ ≈ 86%, *D*_p_ ≈ 1.7 nm) and an alkaline surface chemistry, whereas LP39 combines a very high surface area with a more mesopore-rich pore network (*V*_μ_/*V*_t_ ≈ 32%, *D*_p_ ≈ 3.6 nm) and an acidic, oxygenated surface. These differences are directly reflected in the NMR-derived dynamic signatures. ILT analysis shows that APN1 preserves a largely liquid-like *T*_1_ distribution at high field, with progressive broadening only at lower fields, consistent with partial interfacial exchange and confinement in a micropore-dominated network. In contrast, LP39 induces systematic *T*_1_ shortening already at high field and pronounced distribution reshaping across the entire frequency range, indicating stronger surface-driven relaxation and a larger fraction of dynamically perturbed fluorinated liquid. Quantitative NMRD analysis reinforces this picture: APN1 primarily enhances relaxation efficiency through increased prefactors while retaining bulk-like effective correlation times, whereas LP39 produces strongly dispersive profiles with amplified translational contributions and signatures of restricted diffusion and persistent close-contact configurations. It is important to stress that these trends are consistent across both fluorinated probes (PFD and Galden), demonstrating that the observed effects arise from the biochar texture and surface chemistry rather than from a specific molecular structure of the liquid. Overall, the results show that activated biochars cannot be ranked solely by surface area or uptake capacity; instead, their effectiveness in interacting with fluorinated contaminants is encoded in their ability to redistribute molecular mobility across bulk-like, interfacial, and confined dynamic states. The dynamic-signature approach adopted here therefore provides a mechanistically grounded complement to conventional adsorption metrics and offers a route toward rational, fit-for-purpose selection of biochars for fluorinated-contaminant retention. Direct, quantitative benchmarking of PFAS/organofluorine adsorption uptake for these two specific biochars against the dynamic signatures reported here was outside the scope of the present relaxometry-focused study and represents a natural next step, to be pursued using established batches or column methodologies.^5–8^
